# Supplementary material for: FERN – a Java framework for stochastic simulation and evaluation of reaction networks
Source: BMC Bioinformatics. 2008 Aug 29;9:356. doi: 10.1186/1471-2105-9-356 (PMC2553347; doi:10.1186/1471-2105-9-356)
Supplement: Additional file 1 — FERN distribution, Version 1.3. This archive contains the FERN source code and binaries as well as documentation and example models in FernML and SBML. [file 1471-2105-9-356-S1.zip › fern/doc/javadoc/fern/simulation/algorithm/DependencyGraph.html]

DependencyGraph


---


|  |  |  |
| --- | --- | --- |
| **Overview** \| **Package** \| **Class** \| **Use** \| **Tree** \| **Deprecated** \| **Index** \| **Help** | |  |
| **PREV CLASS**   **NEXT CLASS** | **FRAMES**    **NO FRAMES**     **All Classes** |  |
| SUMMARY: NESTED \| FIELD \| CONSTR \| METHOD | DETAIL: FIELD \| CONSTR \| METHOD |  |


---


## fern.simulation.algorithm Class DependencyGraph

```
java.lang.Object
  fern.simulation.algorithm.DependencyGraph
```

---

``` public class DependencyGraph extends Object ```

Implements a dependency graph. It is a directed graph whose vertices are the reactions
of a network. From vertex i to vertex j is an edge iff i=j or there is at least one species
that changes quantity when reaction i fires and is reactant of reaction j.

This definition implies that if a reaction i fires, the propensities of each descendant
in the dependency graph has to be recalculated and no other.

For reference see M.A.Gibson and J.Bruck, Efficient Exact Stochastic Simulation of Chemical
Systems with Many Species and Many Channels, J.Phys.Chem.A., Vol 104, no 9, 2000

**Author:**
:   Florian Erhard

**See Also:**: `GillespieEnhanced`, `GibsonBruckSimulator`

---

**Constructor Summary**

`DependencyGraph(Network net)`
  
          Creates the dependency graph for a given network


| **Method Summary** | |
| --- | --- |
| `LinkedList<Integer>` | `getDependent(int reaction)`             Gets the descendants of a reaction in the dependency graph |

**Methods inherited from class java.lang.Object**

`clone, equals, finalize, getClass, hashCode, notify, notifyAll, toString, wait, wait, wait`

**Constructor Detail**

### DependencyGraph

```
public DependencyGraph(Network net)
```

:   Creates the dependency graph for a given network

    **Parameters:**: `net` - the network


**Method Detail**

### getDependent

```
public LinkedList<Integer> getDependent(int reaction)
```

:   Gets the descendants of a reaction in the dependency graph

    :   **Parameters:**: `reaction` - index of the reaction **Returns:**: list of dependent reactions


---


|  |  |  |
| --- | --- | --- |
| **Overview** \| **Package** \| **Class** \| **Use** \| **Tree** \| **Deprecated** \| **Index** \| **Help** | |  |
| **PREV CLASS**   **NEXT CLASS** | **FRAMES**    **NO FRAMES**     **All Classes** |  |
| SUMMARY: NESTED \| FIELD \| CONSTR \| METHOD | DETAIL: FIELD \| CONSTR \| METHOD |  |


---
